# Effect of electroporation medium conductivity on exogenous molecule transfer to cells *in vitro*

**DOI:** 10.1038/s41598-018-38287-8

**Published:** 2019-02-05

**Authors:** Paulius Ruzgys, Milda Jakutavičiūtė, Ingrida Šatkauskienė, Karolina Čepurnienė, Saulius Šatkauskas

**Affiliations:** Vytautas Magnus University, Faculty of Natural Sciences, Vileikos 8, Kaunas, Lithuania

## Abstract

In this study we evaluated the influence of medium conductivity to propidium iodide (PI) and bleomycin (BLM) electroporation mediated transfer to cells. Inverse dependency between the extracellular conductivity and the efficiency of the transfer had been found. Using 1 high voltage (HV) pulse, the total molecule transfer efficiency decreased 4.67 times when external medium conductivity increased from 0.1 to 0.9 S/m. Similar results had been found using 2 HV and 3 HV pulses. The percentage of cells killed by BLM electroporation mediated transfer had also decreased with the conductivity increase, from 79% killed cells in 0.1 S/m conductivity medium to 28% killed cells in 0.9 S/m conductivity medium. We hypothesize that the effect of external medium conductivity on electroporation mediated transfer is triggered by cell deformation during electric field application. In high conductivity external medium cell assumes oblate shape, which causes a change of voltage distribution on the cell membrane, leading to lower electric field induced transmembrane potential. On the contrary, low conductivity external medium leads to prolate cell shape and increased transmembrane potential at the electrode facing cell poles.

## Introduction

The barrier function of cell membrane is essential for cell survival. However, there are various reasons in bio-industry and medical treatments that require temporary increase of cell membrane permeability^1,2^. Electroporation, introduced over 40 years ago, is one of the possible methods used to achieve this objective. Phenomenon of electroporation occurs after the application of appropriate electrical field on cells^3^. It is predicted that external electrical field causes transmembrane potential to reach values over the threshold, which triggers the increase of cell membrane permeability^4,5^. Majority of publications agree on the hypothesis that the changes in membrane permeability are caused by formation of pores^6^, thus leading to the term “electroporation”^7^. Pore formation success is dependent on electric field parameters, cell size and the characteristics of external medium^8^. Therefore, electrical fields with different voltages must be used to obtain the same electroporation results on different sized cells or cells in different electroporation media. Evaluation of these electrical field changes requires knowledge of electroporation mechanisms. Although the main principles governing electroporation and electroporation mediated transfer mechanisms are already known or predicted^9^, some details are not clear^10^. One of these details is the influence of external cell medium conductivity.

According to the published electroporation models, medium conductivity alters the effect of membrane permeability increase. However, the impact on membrane electroporation is predicted to be negligible^8,11–13^.

It is notable that electroporation is only the initial process required to achieve molecule transfer. After cell membrane electroporation, small molecule delivery mainly occurs due to the triggered process of diffusion^14,15^. Electrophoresis also has an effect to small molecule transfer, but it is only observed on charged molecules and only during the application of electric fields^15^. Electro-osmosis, as a secondary effect of electrophoresis or ionophoresis, can also influence electroporation mediated molecule transfer^16^. Also it is hypothesized that absorption and convection may have effect on small molecule transfer after electroporation^17^.

It is worth noting that there still is no clear proof of the exact role electroporation medium conductivity plays in mechanisms of small molecule transfer through electroporated membrane. There are a few studies that show the influence of external medium conductivity on molecule electroporation mediated transfer^14,18–25^. Yet, the results from these are controversial. Several published studies show that molecule transfer effectiveness is inversely proportional to external medium conductivity^8,14,18,20,23,25^. Others indicate that molecule transfer efficiency is higher when electroporation medium conductivity is higher^24^. There are also publications which show that cell viability decreases in higher conductivity external electroporation medium^18^. Other published data declares that both cell viability and small molecule transfer decreases in higher conductivity electroporation medium^19^. The amount of published controversial data itself demonstrates the need to resolve the role external electroporation medium conductivity in the mechanism of small molecule electroporation mediated transfer.

In present study, we investigate the influence of external medium conductivity on small molecule transfer through electroporated membrane. Laboratory made neutral pH sucrose based isosmotic electroporation media with conductivity range from 0.0125 S/m to 0.9 Sm was chosen for this investigation. The exogenous molecules chosen for transfer were bleomycin and propidium iodide. Obtained results show that the electroporation mediated transfer of bleomycin and propidium iodide is inversely dependent on medium conductivity. Increased external medium conductivity decreases quantity of the exogenous molecules in the cell but does not decrease percentage of cells with successful molecule transfer when high amplitude electric pulses are used. All results go in agreement with majority of electroporation models and published *in vitro* experiments.

## Methods

### Cell culture and electroporation media

Chinese Hamster Ovary (CHO, average radius 9.7 µm) cells were cultured in Dulbecco’s Modified Eagle Medium (DMEM) (Sigma, D5546) supplemented with 10% Foetal Bovine Serum (FBS) (Sigma, F7524), 1% L-glutamine (Sigma, G7513) and 1% penicillin-streptomycin solution (Sigma, P0781). Cells were passed every 2–3 days and always a day before the experiment.

Electroporation media were made with compositions shown in Table 1. Conductivity and pH were measured to ensure that pH is neutral (~7) and conductivity is correct. All the media had 270 mOsm/l osmolarity (isoosmotic), to neglect the possible role of osmotic pressure on small molecule electroporation mediated transfer. After the preparation of solutions, media were sterilized by filtering (filter pore radius 0.22 μm).

**Table 1:** Composition of electroporation media.

	Na_2_HPO_4_, mM	NaH_2_PO_4_, mM	MgCl_2_, mM	Sucrose, mM
EP medium 1 (0,0125 S/m)	0.70	0.375	0.22	266.70
EP medium 2 (0,025 S/m)	1.40	0.75	0.43	263.39
EP medium 3 (0,075 S/m)	4.19	2.25	1.30	249.03
EP medium 4 (0.1 S/m)	5.59	3.00	1.73	242.19
EP medium 5 (0.3 S/m)	16.81	9.04	5.21	186.30
EP medium 5 (0.5 S/m)	28.31	15.23	8.77	129.06
EP medium 7 (0.7 S/m)	39.05	21.00	12.10	75.80
EP medium 8 (0.9 S/m)	50.88	27.34	15.76	16.74

### Process of experiment

Propidium iodide (PI) with final concentration of 40 µM and bleomycin (BLM) with final concentration of 20 nM were used as reporter molecules. From 1 to 3 high voltage (HV) electric pulses (1200 V/cm amplitude, 100 μs duration, 1 Hz frequency) were triggered using electroporator constructed in Kaunas University of Technology and Vytautas Magnus University. 50 µl of cell suspension, containing 9 × 10^4^ cells and aforementioned concentration of reporter molecules, was put between stainless steel plate electrodes with 2 mm distance between the electrodes. Appropriate electric fields were applied immediately.

Experiments conducted using BLM or just electric pulses were done as follows. After electric field application, cells were placed in 24 well plate for 10 minutes, then diluted with DMEM complete medium. 400 cells were taken and put to 2 ml of DMEM complete medium in 40 mm Petri dish and incubated for 6 days. Then cells were fixed with 70% ethanol for 10 minutes and stained with 10% crystal violet solution.

Experiments conducted using PI after electroporation were done as follows. Cells were gathered into 1.5 ml Eppendorf tubes. 10 min after electroporation cells were diluted with 100 μl of appropriate conductivity electroporation medium and measured using flow cytometer.

In pore resealing dynamics experiments, PI was not present in the electroporation medium during electroporation, except for positive control. Instead, cells were electroporated in PI free medium, and PI was added 0, 20, 60, 120, 300, 600 and 1200 seconds after the electric field application.

### Flow cytometry measurements

**Figure 1:**
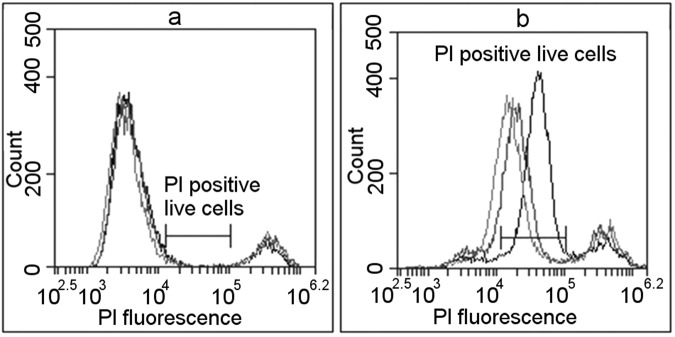
The definition of successful PI electroporation mediated transfer using flow cytometry measurements. Black lines represent used electroporation medium conductivity of 0.1 S/m, red – 0.5 S/m, green 0.9 S/m. Blue marked area defines the fluorescence limits considered to represent successful PI electroporation mediated transfer; it is marked as “PI positive live cells”. (**a**) Shows cells without electrical pulse application. Peak with lower fluorescence represents unaffected cells (autofluorescence), peak with high fluorescence represents dead cells (highly permeable to PI). The area between peaks is defined as area where live PI positive cells would occur after successful PI transfer. (**b**) Shows cells after PI electroporation mediated transfer in different conductivity media after the electric impulse application. The majority of cells moved from first (autofluorescence) peak with low fluorescence (unaffected cells) to the area marked as “PI positive live cells”.

BD Accuri C6 flow cytometer was used in flow cytometry measurements. For evaluation of PI electroporation mediated transfer, cells were excited using 488 nm laser and the fluorescence was collected using FL2 (585/40) filter. It is known that PI stains dead cells^26^. It is also known that small number of cells does not survive trypsinisation. Therefore, population of control cells (PI, no electroporation) have two PI fluorescence peaks in the FL2 histogram (Fig. 1a). The wider peak with lower fluorescence shows autofluorescence of viable cells, and the second peak with higher fluorescence dead cells with PI fluorescence. PI fluorescence after successful transfer to the live cells is higher than autofluorescence and, as our results, show lower than dead cell fluorescence (Fig. 1b). We consider that cells with successful PI transfer have fluorescence values between cell autofluorescence and PI fluorescence in dead cells. In Fig. 1, this area is marked as “PI positive live cells”.

### Visualisation of electroporation

For visualisation experiments, cells were grown on glass cover slips in a 40 mm radius Petri dish. Copper electrodes with 2 mm gap between the electrodes were used for electroporation. Electroporation media of 0.1 S/m conductivity was used. 1 HV (720 V/cm, 100 µs) pulse was delivered to electroporate the cells. Cells were imaged using Motic AE31 fluorescent microscope, and images were taken using MoticamPro 285B camera with Motic Images Advanced 3.2 software. For each experimental point, brightfield image and a series of fluorescent images (D560/40X excitation, dichroic 595DCLP mirror, D630/60 emission) were taken, with the images in fluorescent series taken every 1 sec, 5 seconds before the application of the pulse and monitored for 60 seconds after the electric pulse. The images were processed using open-source software ImageJ to calculate Corrected Total Cell Fluorescence. The calculations were done as described in^27^. Shortly, the cell area was calculated from brightfield pictures, and the cell area was multiplied with the average fluorescence in cell area in the time-series of fluorescent images, thus enabling the monitoring of PI entry dynamics.

### Statistics

All experiments were performed with three repetitions per experiment, with at least two separate experiments conducted on different days. For visualisation, 100 cells per field of view were used to calculate the average CTCF. Two-tailed T-test for independent samples was used to test for significance. Throughout the rest of the article, *signifies p values < 0.05, **signifies p values below 0.01.

## Results and Discussion

**Figure 2:**
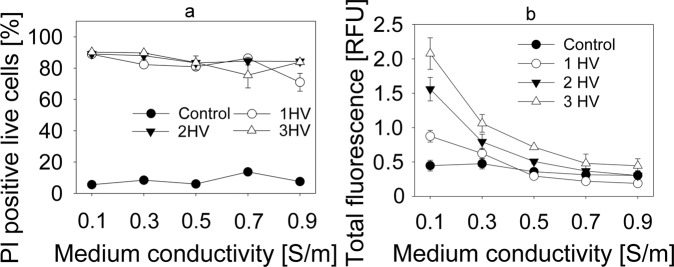
PI electroporation mediated transfer to CHO cells in electroporation media with different conductivities. The fluorescence differentiation between cells that are dead, unaffected, and successfully loaded with PI is described in Fig. 1. (**a**) Shows the percentage of cells with successful PI electroporation mediated transfer in different conductivity electroporation media. (**b**) Shows the mean relative PI fluorescence.

Flow cytometry was first used to determine the dependence of propidium iodide (PI) electroporation mediated transfer on external medium conductivity. The dependence of live PI positive cell percentage on electroporation medium conductivity is shown in Fig. 2a. The mean PI fluorescence in the live cells with successful PI transfer is shown in Fig. 2b. Figure 2a does not show any differences in the percentage of PI positive cells when using electroporation media with different conductivities. However, Fig. 2b clearly indicates that mean cell fluorescence decreases when cells are electroporated in higher conductivity electroporation medium. This allows the assumption of that these cells have lower quantities of PI inside. Same results are seen with all numbers of electric pulses used, although additional HV pulses do increase mean cell fluorescence in electroporation media that have conductivities lower than cytoplasm conductivity, which is estimated to be 0.5 S/m^28,29^. Mean cell fluorescence in cells electroporated with 1 HV pulse decreased 4.67 times when comparing between 0.1 S/m and 0.9 S/m electroporation media. Comparison between 0.1 S/m and 0.9 S/m electroporation media in cells electroporated using 2 HV and 3 HV electric pulse sequences yielded similar results. In all used electroporation sequences, highest mean fluorescence drop was observed in conductivity range from 0.1 S/m to 0.5 S/m.

**Figure 3:**
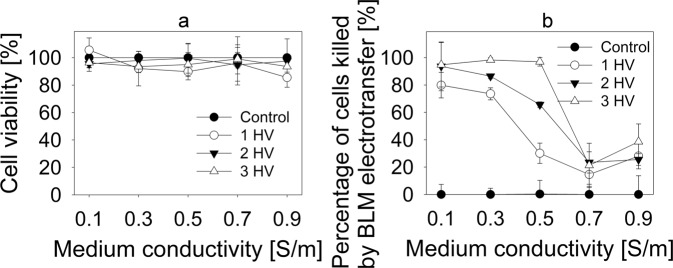
Bleomycin electroporation mediated transfer to CHO cells in electroporation media with different conductivities. (**a**) Shows the percentage of killed cells due to electroporation only in different conductivity electroporation media. (**b**) Shows the percentage of cells killed due to bleomycin electroporation mediated transfer only in different conductivity electroporation media. Data in (**b**) data was obtained by subtracting the percentage of dead cells after electroporation alone from the percentage of dead cells after bleomycin electroporation mediated transfer.

These findings led to a decision to observe the limiting effect of higher electroporation medium conductivity on *in vitro* model of electrochemotherapy. Anticancer drug bleomycin (BLM) was chosen as it is most widely used in clinical practice in electrochemoterapy^30^. Results from these experiments are seen in Fig. 3. Figure 3a shows that no significant difference in cell viability after electroporation without any drug is observed when conductivity of electroporation media is changed. However, electroporation medium conductivity had substantial effect on BLM electroporation mediated transfer. Results are calculated, taking a percentage of cells killed by BLM and electroporation and subtracting the percentage of cells killed by electroporation alone, thus allowing to evaluate only fraction of cells killed by the action of BLM. Obtained data is presented in Fig. 3b. When 1 HV pulse was used in 0.1 S/m conductivity media, 79% of cells were killed as a result of BLM electroporation mediated transfer. In 0.3 S/m medium, BLM electroporation mediated transfer resulted in 73% of cells killed. When electroporation medium conductivity was increased to 0.5 S/m, percentage of killed cells because of BLM electroporation mediated transfer decreased to 30%. In 0.7 S/m and 0.9 S/m conductivity media BLM electroporation mediated transfer resulted in 15 and 28% of killed cells, respectively. Using 2 HV pulse combination, the highest decrease of killed cells was observed in 0.5 and 0.7 S/m conductivity media: from 93% of cells killed because of BLM electroporation mediated transfer in 0.1 S/m conductivity media, the percentage of killed cells decreased to 65% and 23%, respectively. It is interesting to note that using 3 HV pulse combination, the fall range diminished only between 0.5 and 0.7 S/m conductivity media. The percentage of killed cells decreased from 96% in 0.5 S/m electroporation medium to 21% in 0.7 S/m conductivity electroporation medium.

It is also notable that lowest BLM transfer efficiency with all pulse combinations was in 0.9 S/m conductivity electroporation medium and did not differ between pulse combinations. Experiments with BLM clearly indicate inversely proportional effect of electroporation mediated transfer efficiency in dependence of external medium conductivity.

After making these experimental observations, we decided to investigate the theoretical basis of these findings. There are a few processes that can impact the molecular transport after electroporation, one of them being the efficiency of pore formation. However, the current literature does not report any significant differences in the pore formation in different conductivity external media. Our results also support these observations as the percentage of successfully electroporated cells did not change with conductivity of electroporation medium (Fig. 2a), but the transport of small molecules significantly decreased with increasing medium conductivity (Figs 2b and 3b).

While pore formation is not a cause for different small molecule electroporation mediated transport in different conductivity media, pore size and resealing mechanics might have an impact on it. Tekle *et al*. suggested that pores reseal faster in higher conductivity electroporation medium^31^. Higher ion inflow will be observed with higher conductivity of electroporation medium, leading to higher rate of interaction with intracellular counter ions and local diminishment of transmembrane potential. Lowered transmembrane potential leads to increase in pore surface tension and thus the shrinking of the pore^32^. Reduction in pore radius could explain the results in Fig. 2a,b, where we can see the reduction in the fluorescence intensity, but not the percentage of PI positive living cells.

**Figure 4:**
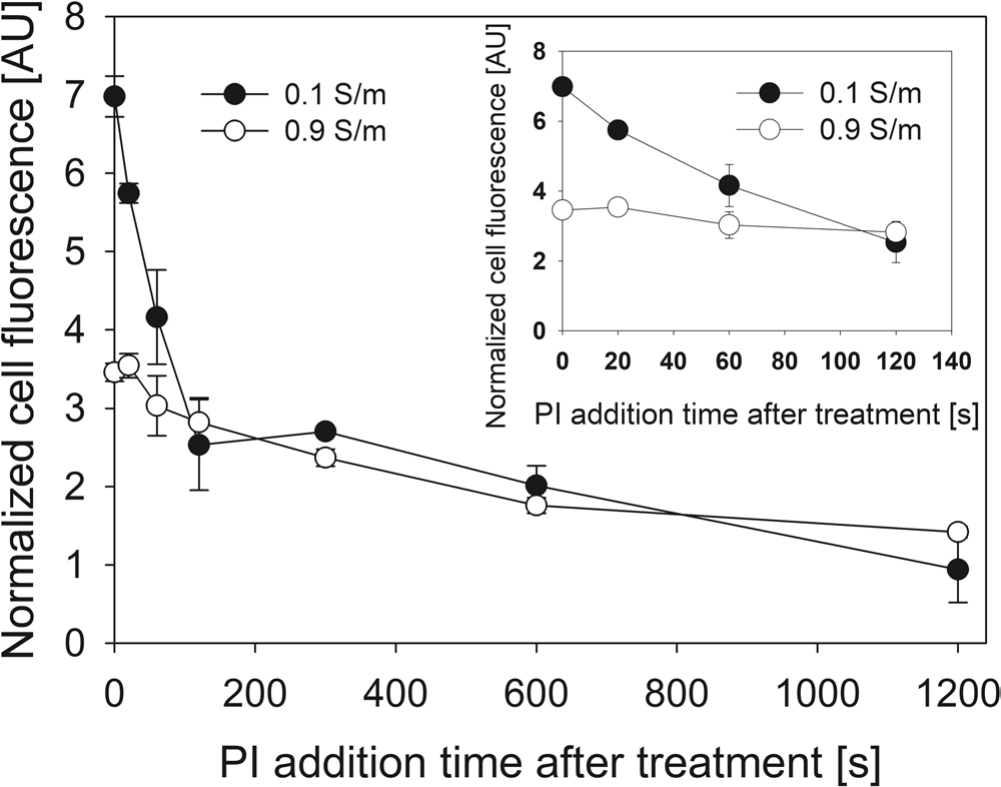
Dependence of pore resealing time on conductivity of electroporation medium. The fluorescence of PI positive live cells was determined as described in Fig. 1. The results use the fluorescence normalized to that of control (not affected by electric field) cell fluorescence.

To test this, we electroporated the cells and added PI on set times after the electroporation (Fig. 4). These results were normalized to the fluorescence of control (no electroporation) cells. When PI was added up to 60 seconds after the HV delivery, the normalized PI fluorescence was higher in 0.1 S/m conductivity medium, signifying higher PI transfer. However, when PI was added more than 60 seconds after the delivery of HV, there were no significant differences between normalized transfer in 0.1 S/m and 0.9 S/m media. However, the decay in fluorescence is observed up to 1200 seconds (20 minutes) after electroporation. This shows that initial stages of pore resealing are slower in low conductivity medium, however, the later stages happen independently of medium conductivity. Similar idea was proposed in^32^, where the authors stated that final stage of pore resealing was dependent on the reordering of phospholipids in the membrane and not related to the parameters of electric field.

Another possible reason of lower small molecule transport in higher conductivity medium is related to a phenomenon called Born’s electrostatic energy restriction. As molecules need to go through the electropores lined with charged phospholipid heads, the walls of the pore will interact with the ions flowing through. This will cause a loss of ion energy, and thus will reduce the ion movement speed^33^. As Born’s electrostatic energy restriction increases with the charge number^34^, the ion slowing effect will be higher in higher conductivity medium.

Alternative explanation for reduction of small molecule electroporation mediated transport in high conductivity external medium was proposed by^13,35^. It relies on the phenomenon called Field Amplified Sample Stacking (FASS). The effect of FASS is used in separation sciences and it causes the change in molecule movement velocity when moving from lower conductivity medium to higher conductivity medium^36^. As molecule movement speed is faster in high-conductivity medium and lower in low-conductivity medium, it may cause the molecules of interest to concentrate (“stack”) at the pore-cytoplasm interface if the conductivity of external medium is lower than the cytoplasm^13,35^.

**Figure 5:**
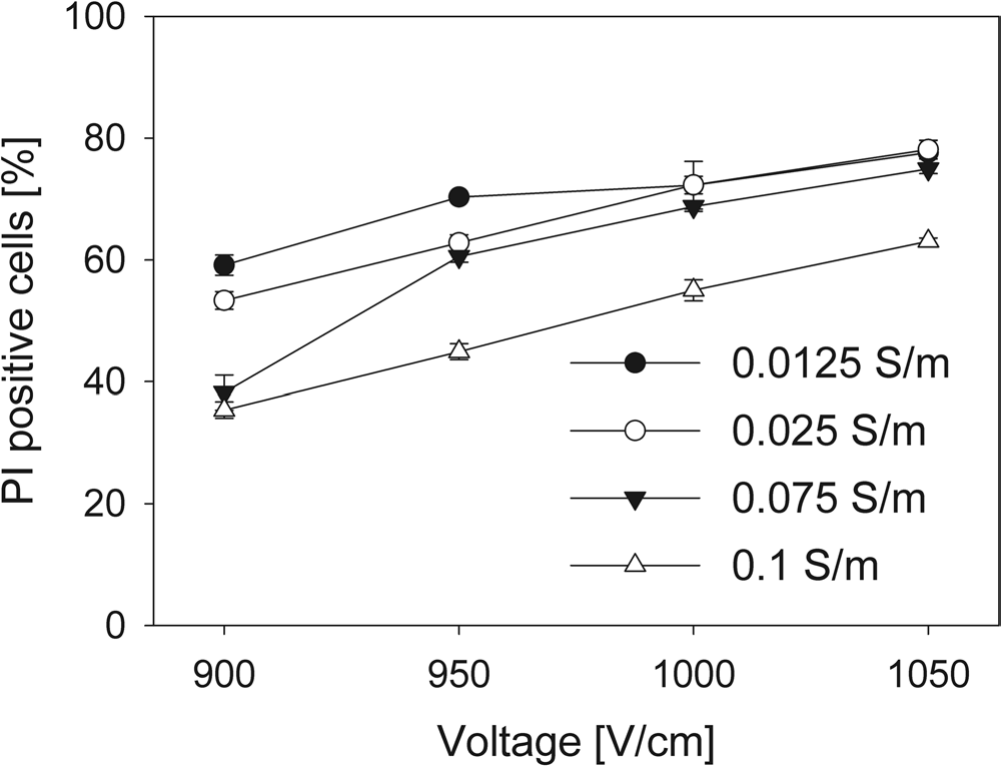
PI electroporation mediated transfer to CHO cells in electroporation media with different conductivities. The fluorescence differentiation between cells that are dead, unaffected, and successfully loaded with PI is described in Fig. 1. The results show the percentage of cells with successful PI transfer in different conductivity electroporation media.

However, it should be noted that model described in^13^ assumes that the electroporation threshold is the same between media of different conductivities. Indeed, the results of these authors’ modelling show no significant differences in electric field induced transmembrane potential between media of 0.1 and 0.9 S/m conductivities. Similarly, our results showed no differences of the percentage of electroporated cells in different conductivity media (Fig. 2a). We expanded these results with media of very low conductivities along the range of electric pulse voltages. Surprisingly, our results show that the threshold electric field values to induce PI electroporation mediated transfer were indeed higher with higher electroporation medium conductivities (i.e., higher electric field was needed to cause the same amount of PI positive cells). When lower pulses were used, the percentage of PI positive cells was lower in higher conductivity media as seen in Fig. 5. With 1200 V/cm, there is no significant difference between the percentage of PI positive cells anymore, but the fluorescence of PI positive cells is still higher in lower conductivity medium (Fig. 2). Seemingly, the conditions used in our original experiments had the conditions for plateau values of percentage of PI positive live cells. As seen in Fig. 2a, the same amount of electroporated cells were obtained with all the conductivities of electroporation medium, when used electric pulses were significantly higher than the threshold for electroporation.

One of the possible reasons for this behaviour is the phenomenon of cell deformation in electric fields. The differential deformation of lipid vesicles that had sizes comparable to those of eukaryotic cells in pulsed electric field was described by Dimova *et al*.^37^. When the conductivity inside the vesicle was greater than the external conductivity, the vesicles would become prolate or cigar shaped (i.e., the spheroid radius along the direction of electric field would be larger than the radius perpendicular to the direction of electric field) upon the application of electric field. Inversely, if the conductivity inside the vesicle were lower than external conductivity, the vesicles would become oblate or disk shaped (i.e., the spheroid radius along the direction of electric field would be smaller than the radius perpendicular to the direction of electric field). If both conductivities were the same, vesicles would remain spherical^37^. The authors observed these effects using electric field with frequencies ranging from 1 kHz to 1 MHz and durations ranging from 50 µs to 300 µs. The vesicles were also reported to return to spherical form after 50 ms^37^. That allows us to assume that pulses with frequency lower than 20 Hz would have effect similar to independent DC pulses. According to the same authors, oblate and prolate deformations were observed using DC pulses as well^37^.

**Figure 6:**
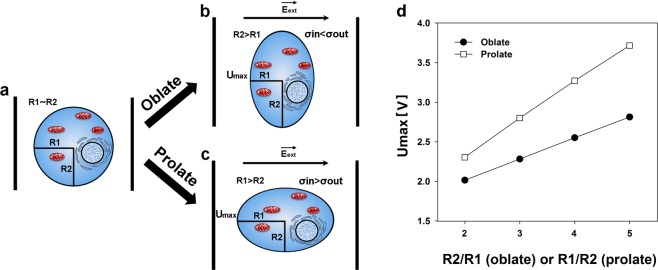
Graphical representation of cell deformation impact on electroporation. (**a**) Shows the cell before the application of electric field. Cell radius parallel to the direction of electric field (R1) is approximately equal to the cell radius perpendicular to the direction of electric field (R2). During the application of electric fields, R1 will become lower than R2 (oblate shape) if medium conductivity is higher in extracellular medium in comparison to the cytoplasm (σ_in_ < σ_out_) (**b**). Similarly, R1 will become larger than R2 (prolate shape) if medium conductivity is lower in extracellular medium in comparison to the cytoplasm(σ_in_ > σ_out_) (**c**). In both of these cases, the cross-section area would remain spherical. This deformation is caused by Maxwell stress and the effect of tangential electrical field. Due to this, area affected by electric field affected will become larger in oblate cell, and smaller in prolate cell. (**d**) Shows the maximum induced transmembrane potential (induced transmembrane potential at cell poles) on a 9.7 µm radius cell, deformed and electroporated by a single 1200 V/cm electric pulse. It can be seen that for the same deformation degree, the prolate spheroids always have higher maximum induced transmembrane potential than the oblate spheroids.

We propose that the cells in aforementioned conditions would deform similarly to cell-sized vesicles, as summarized in Fig. 6. The pulses used in our experiments would either cause the cells to become prolate or oblate, depending to external medium conductivity.

This means that the cell radius changes in two directions: perpendicular to the direction of electric field and parallel to the electric field (note that the cross section of the cell on the plane parallel to the electrodes remains circular). As one of these radii shortens, the other elongates, i.e., the changes in cell radii lengths are inverse. It should be noted that the cross section of the cell parallel to electrodes would remain spherical, leading to a smaller area of cross section on a prolate spheroid and a larger area of cross section on an oblate spheroid.

The basic equation used to calculate the induced transmembrane potential in a sphere-shaped object is called Schwan Equation (see Eq. 1)^38^. According to this equation, the induced transmembrane potential (U) is calculated by multiplying external electric field value (E) with cell radius (R) and angle (cos (θ)) between the electric field vector and the membrane normal vector at the site on the membrane where transmembrane potential is measured.

1 $$U\,=\,1.5\,ER\,cos\,\theta $$

However, this equation is only valid for sphere shaped objects, and not oblate or prolate spheroids. To calculate the changes in transmembrane potential for these, another solution is needed. Kotnik and colleagues^38^ described a method to calculate the transmembrane potential for oblate and prolate spheroids, where Laplace’s equation is solved in a suitable coordinate system including perpendicular and parallel cell radii. The model proposed by these authors includes different calculations for oblate and prolate spheroid geometries. Equation 2 is valid for oblate spheroid with the axis of rotational symmetry aligned with the direction of electric field.

2 $$U\,=\,E\frac{{R}_{2}^{2}-{R}_{1}^{2}}{\frac{{R}_{2}^{2}}{\sqrt{{R}_{2}^{2}-{R}_{2}^{1}}}arcctg\frac{{R}_{1}}{\sqrt{{R}_{2}^{2}-{R}_{2}^{1}}}-{R}_{1}}\times \frac{{R}_{2}\,{\cos }\,{\rm{\theta }}}{\sqrt{{R}_{1}^{2}{si}{{n}}^{2}{\rm{\theta }}+{R}_{2}^{2}\,{\cos }\,{\rm{\theta }}}}$$

Equation 3 is used for the evaluation of transmembrane potential induced in prolate spheroid with the axis of rotational symmetry aligned with the direction of electric field.

3 $$U\,=\,E\frac{{R}_{1}^{2}-{R}_{1}^{2}}{{R}_{1}-\frac{{R}_{2}^{2}}{\sqrt{{R}_{1}^{2}-{R}_{2}^{2}}}\,\mathrm{ln}\,\frac{{R}_{1}+\sqrt{{R}_{1}^{2}-{R}_{2}^{2}}}{{R}_{2}}}\times \frac{{R}_{2}\,{\cos }\,{\rm{\theta }}}{\sqrt{{R}_{1}^{2}{si}{{n}}^{2}{\rm{\theta }}+{R}_{2}^{2}\,{\cos }\,{\rm{\theta }}}}$$

In Eqs 2 and 3, R1 and R2 are cell radii perpendicular to and parallel to the electric field, respectively (see Fig. 6). Both of the radii, as well as the value of external electric field (E) and the angle (θ) between the electric field vector and the membrane normal vector at the site on the membrane where transmembrane potential is measured are used to calculate the induced transmembrane potential (U) in these cases.

These models show that differences in induced transmembrane potential between spherical and non-spherical cells are based not only on the change in radius parallel to the electric field (R1), but also on the different modes of transmembrane potential distribution along the membrane. According to the authors, only a small region of the membrane of prolate cells has induced transmembrane potential that is close to the maximum value. However, in oblate cells, this region occupies most of the membrane^39^. In our case, the cell in high conductivity medium would have larger membrane area affected by electric field in comparison to the cells with same resting radius in low conductivity medium.

We used the Eqs 2 and 3 to calculate the maximum induced transmembrane potential, Umax (i.e., the transmembrane potential at the cell poles facing the electrodes) for the prolate and oblate spheroids that are formed from the cells with the same resting radius due to external conductivity dependent electrodeformation. For this approximation, we assumed the resting cell radius was 9.7 µm (average radius of CHO cells), and the external electric field used to trigger electroporation and electrodeformation was set to 1200 V/cm, same as the one used in the bulk of experiments described above. Constant cell volume was assumed when calculating the lengths of radii R1 (parallel to the electric field) and R2 (perpendicular to electric field). We calculated the radii R1 and R2 for oblate and prolate spheroids with R2/R1 or R1/R2 ratios respectively ranging from 2/1 to 5/1. The results of these calculations are presented in Fig. 6d. One can see that for the same degree of deformation, Umax is always higher for the prolate spheroids than for the oblate spheroids. For the aspect ratio 3/1, the Umax is ~0.5 V higher in the case of prolate deformation. For the aspect ratio 5/1 (same as the ones Kotnik *et al*.^38^ used to develop the formulae used), the Umax is ~0.9 V higher in the case of prolate deformation.

This allows us to conclude that these considerable changes on the transmembrane potential intensity, as well as their areal distribution, has a significant impact on the electroporation process, and consequently to the process of small molecule electroporation mediated transfer.

**Figure 7:**
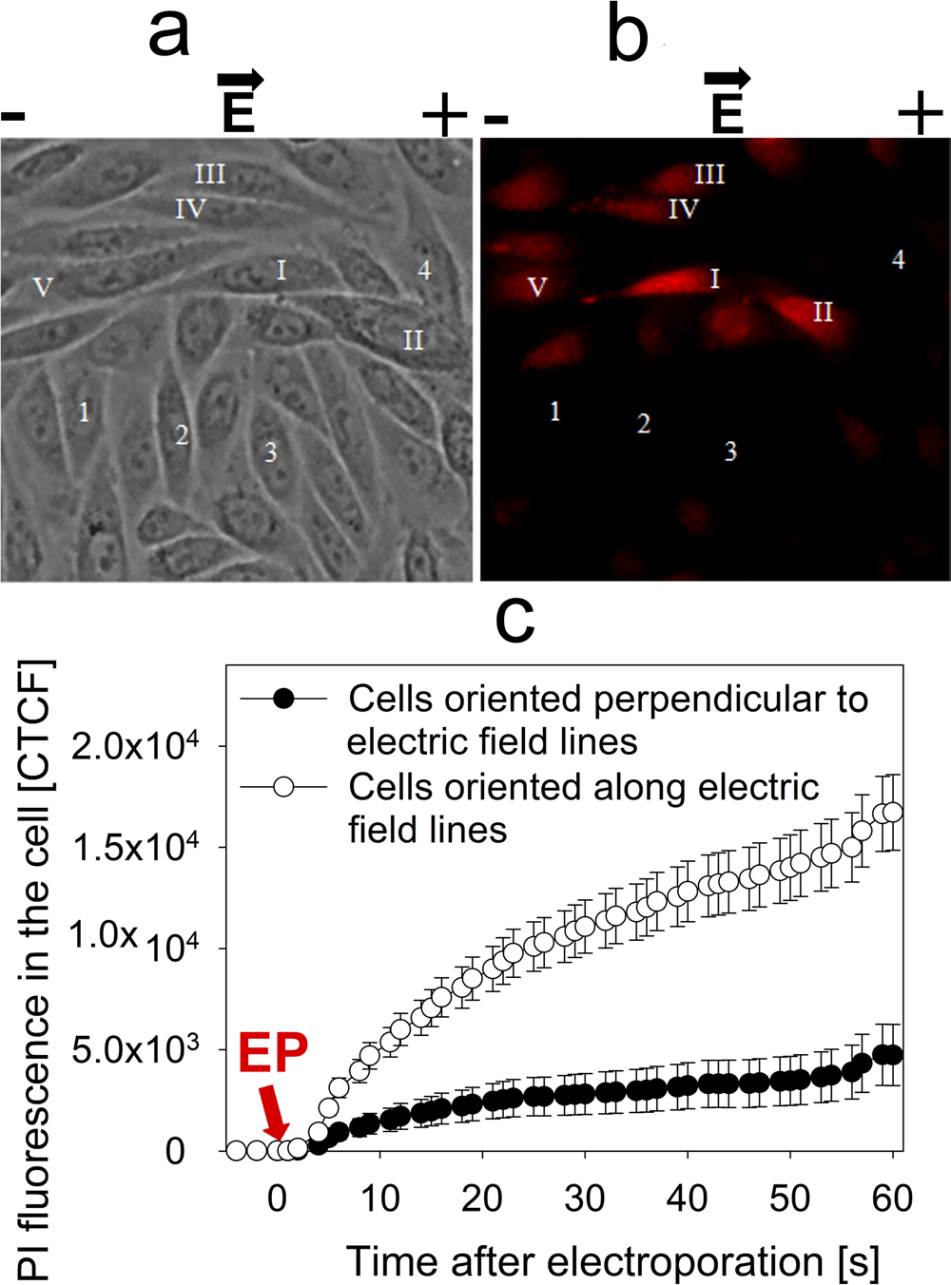
Fluorescence intensity of adherent cells oriented perpendicular and along the electric field lines, visualized under fluorescent microscopy. (**a**) Shows cells before the application of electric field, (**b**) shows the same cells after the application of electric field. It can be visually observed that cell fluorescence is higher in cells oriented along the electric field lines (I-V) in comparison to the cells oriented perpendicular to the electric field lines (1–4). These results are numerically represented in (**c**), where corrected total cell fluorescence was calculated by multiplying the average fluorescence in the cell with the cell area. The changes in cell fluorescence were monitored 5 seconds before the application of electric pulse and 60 seconds after. The arrow shows the time of electroporation.

In order to experimentally confirm this theory, we conducted experiments, visualising PI transfer by electroporation to adherent cells that are aligned with their longer axis situated along or perpendicular to electric field lines. Our results clearly show that cells with their longer axis parallel to the direction of electric field have higher PI electroporation mediated transfer in comparison to the cells with their longer axis perpendicular to the direction of the electric field (Fig. 7). Even more, the dynamics of PI electroporation mediated transfer in the period of 60 seconds after the electric field application (Fig. 7c) indicate lesser PI transfer after electroporation. Such results indicate that lesser area of the cell membrane is electroporated when cells are oriented along the electric field in comparison to the cells oriented perpendicular to electric field. Similar results have already been demonstrated by Phez and colleagues^40^.

With this, we can also deduce that the results in Fig. 4 are caused by the different size of electroporated area, depending on the ratio of inner and outer cell conductivity. The pore resealing dynamics indicate that this significantly higher electroporated area of the membrane becomes insignificant 60 s post electric field effect.

In summary, we show the inverse dependence of small molecule (PI and BLM) electroporation mediated transfer to cell and external medium conductivity. We also propose hypothesis that increase in external medium conductivity causes partial blockage of electropores for PI and BLM due to Born’s electrostatic energy restriction and instability of small electropores induced by higher ion flow triggered current, or by decreased transmembrane potential. In parallel, we suggest that cell deformation process induced by high external medium conductance decreases transmembrane potential. As a result, electropores or electroporated area becomes smaller, thus disabling efficient PI and BLM transfer by electroporation.
